# Edible Coating Based on *Konjac glucomannan* Loading *Ocimum gratissimum* Essential Oil for Postharvest Preservation of *Orange*

**DOI:** 10.3390/polym17091217

**Published:** 2025-04-29

**Authors:** Xiang Yu, Jingyu Zhu, Jintao Wu, Yuhang Cheng, Ya Gao, Yi Liu, Fatang Jiang

**Affiliations:** 1 Cooperative Innovation Center of Industrial Fermentation (Ministry of Education & Hubei Province), Hubei University of Technology, Wuhan 430068, China; yuxiang20010410@163.com (X.Y.); zhujingyu12355@163.com (J.Z.); wujintaozzw@163.com (J.W.); jiangft@mail.hbut.edu.cn (F.J.); 2 Glyn O. Phillips Hydrocolloid Research Centre at HUT, School of Life and Health Sciences, Hubei University of Technology, Wuhan 430068, China; 3 Faculty of Engineering, University of Nottingham, Nottingham NG7 2RD, UK

**Keywords:** *Konjac glucomannan*, edible coating, *Ocimum gratissimum* essential oils, *orange* preservation, fruit quality

## Abstract

Microbial contamination challenges have led to the development of active edible coatings for fruit preservation. Herein, a *Konjac glucomannan* (KGM) coating loaded with *Ocimum gratissimum* (OG) essential oil stabilized by pectin with superior resistance to air permeability, oxidation, and fungal, was prepared in situ on the surface of *Mandarin oranges* to enhance postharvest fruit quality. The results demonstrated that the KGM-pectin-OG (K-P-OG) 1.5 wt% coating exhibited good performance in terms of stability, adhesion, and wetting. Meanwhile, the coating had an ideal air permeability due to its compact and dense structure based on the good compatibility and interactions between the components. The oxygen permeability of the K-P-OG coating was 7.9 × (10^−16^ g·cm)/(cm^2^·s·Pa), which was six orders of magnitude lower than that of the KGM coating. The antioxidant, in vitro, and in vivo antifungal activities against *Penicillium italicum* of the coating were strengthened by the OG emulsion and mainly depended on its concentration. The storage results showed that the K-P-OG 1.5% coating extended the shelf life of *Mandarin oranges* by 8 days, reduced the weight loss rate by 13%, and increased the firmness and POD during storage by 24.14% and 100%, respectively, compared with the control group. These results demonstrate that K-P-OG can effectively maintain nutrient content and extend the storage time of *Mandarin oranges* by enhancing antioxidant capacity and inhibiting fruit respiration and microorganism growth. This study presents a strategy for developing edible coatings for postharvest fruit preservation.

## 1. Introduction

With great economic and health value, citrus is one of the most popular and widely grown fruit crops globally [1,2]. However, citrus fruits are prone to spoilage due to irreversible postharvest microbial erosion (mainly *Penicillium italicum* and *Penicillium digitatum*) caused by their inherent high nutritional content coupled with the external environment, which has caused huge economic losses [3]. Researchers have explored various preservation strategies, such as low-temperature storage, modified atmosphere packaging, irradiation, and treatment with chemical agents, to maintain fruit quality during storage [3]. Among them, edible coatings have gained growing interest as a promising technology. Edible coatings are applied to the surface of fruits by dipping, spraying, brushing, or dripping and can provide a semi-permeable barrier to regulate the exchange of water, solutes, and gases (e.g., carbon dioxide and oxygen) between the internal and external atmospheres [4,5]. Furthermore, edible coatings loaded with natural active components could be a green alternative to postharvest chemical treatments and synthetic fungicides, which pose significant risks to health and the environment. The application of edible coatings has huge potential for extending shelf life and reducing quality losses by delaying the metabolic process and retarding microbial growth.

Biodegradable biopolymers of edible coatings (e.g., polysaccharides, phospholipids, and proteins) are predicted to be completely degradable in nature [6]. The polysaccharide edible coating exhibits excellent gas-selective permeability due to its well-ordered and densely packed hydrogen-bonded network structure. This enables fruits to be stored in modified atmospheres without creating anaerobic conditions [7]. In addition, the high stability, adhesiveness, allergen-free nature, and biocompatibility of polysaccharides make them the most promising candidates for major edible coating-forming materials and an alternative to traditional petrochemical-based plastics [8].

Konjac glucomannan (KGM) is a natural polymer polysaccharide. The primary structure of KGM consists of β-d-mannose and β-d-glucose units linked by β-1, 4-glucoside bonds, with branched chains connected via β-1, 3-glucoside bonds. KGM is extensively utilized for edible coatings due to the flexibility and extensibility conferred by its long-chain structure [9,10]. However, due to the abundance of hydroxyl and carboxyl groups, KGM is highly hydrophilic, which limits its ability to effectively wet and spread across the non-polar waxy surfaces of fruits [11]. Studies have shown that adding natural hydrophobic functional compounds, especially essential oils, can effectively improve the hydrophilic–hydrophobic balance of the film and enhance the barrier performance while leveraging the potent antimicrobial activity of the coatings [11,12].

Ocimum gratissimum (OG) essential oils are plant-derived oily active compounds, the main component of which is eugenol. With particular aromas, it has been widely applied in the food, pharmaceutical, and perfume industries due to its strong antioxidant and antimicrobial activities [13,14]. Eugenol has been shown to inhibit the growth of *Penicillium* remarkably and is an alternative to natural preservatives for citrus [15]. However, poor compatibility, vulnerability to oxidation, and inadequate retention of hydrophobic active ingredients have restricted their application range. Employing encapsulation techniques offers a promising solution for improving the efficiency of these active components [16]. Therefore, High-methoxyl pectin, a plant anionic polysaccharide, was employed as an emulsifier to connect hydrophobic OG and the hydrophilic KGM matrix, considering both encapsulation performance and compatibility with the matrix. On the one hand, it could provide good stabilization ability, contributing to improving the retention rate of the OG [17]. Meanwhile, pectin and KGM are well-compatible due to hydrogen bonding interactions between them, which could benefit the compact and uniform structure [18,19].

This study aimed to prepare KGM-based coatings incorporated with OG for *mandarin orange* postharvest preservation. The OG was encapsulated in pectin and then integrated into a KGM matrix (K-P-OG) to enhance its wetting, barrier, and antifungal properties. The stability, apparent viscosity, and structure of the K-P-OG coating were investigated. Subsequently, the barrier and release properties, as well as the antioxidant, in vitro, and in vivo antifungal activities against *Penicillium italicum*, were discussed. Finally, the efficacy of the coating during storage was assessed by measuring the firmness, weight loss, total acid content, and peroxidase activity of the coated *mandarin oranges*. The present results may contribute to the development of active edible coatings aimed at extending the shelf life of fresh fruit.

## 2. Materials and Methods

### 2.1. Materials

Essential oils of *Ocimum gratissimum* (OG) were procured from Ysenyuan Plant Spices Co., Ltd. (Jian, China). KGM powder was obtained from Konson Co., Ltd. (Wuhan, China). High-methoxyl pectin was procured from Sigma Co., Ltd. (Shanghai, China). All chemical reagents used were of analytical grade.

### 2.2. Fabrication of KGM-Based Coating

KGM and high-methoxyl pectin powder were dissolved in distilled water and stirred overnight to achieve dispersions of 1 wt% KGM and 2 wt% high-methoxyl pectin, respectively. The OG was emulsified using a high-methoxyl pectin dispersion (2 wt%), employing a homogenizer (FA25D, FLUKO Technology Development Co., Shanghai, China) at 19,000 rpm for 3 min at 25 °C. Finally, the OG emulsion was incorporated into the 1 wt% KGM dispersion with stirring to obtain the K-P-OG coating solution. The ratio of KGM to high-methoxy pectin was 6:4. The coating solutions with varying OG concentrations were designated as K-P-OG 0.5%, K-P-OG 1.0%, K-P-OG 1.5% and K-P-OG 2%.

### 2.3. Characterization of Coating Solutions

#### 2.3.1. Stability

The droplet size distribution of the coating solutions was analyzed using a Mastersizer 2000 (Malvern Instruments Ltd., Malvern, UK). The morphology and dispersion state of the emulsion droplets were observed using a confocal laser scanning microscope (CLSM) (Leica Microsystems Inc., Heidelberg, Germany) at 40× magnification with a 488 nm excitation wavelength. The physical stability of the coating solution was analyzed using a Turbiscan stability analyzer (Formulation, Toulouse, France). The samples were scanned every 30 min for 12 h to analyze the Turbiscan Stability Index (TSI) changes.

#### 2.3.2. Apparent Viscosity

Steady shear measurements (0.1 to 100 s^−1^) were conducted using a MARS-60 rheometer (Haake Co., Ltd., Vreden, Germany) equipped with a parallel plate of 20 mm diameter at 25 °C. All tests were carried out in triplicate.

#### 2.3.3. Scanning Electron Microscope (SEM)

The samples were uniformly dispersed on a sample stage coated with a double-sided adhesive and subsequently sprayed with gold powder. The microscopic morphologies of the samples were examined by scanning electron microscopy (JSM-6390LV, JEOL Ltd., Tokyo, Japan).

#### 2.3.4. Air Barrier Property

The oxygen permeability (OP) of the coating films was assessed at 25 °C and 0% relative humidity using an OTR test instrument (Labthink Instrument, Jinan, China), while the water vapor permeability (WVP) of the coating film was assessed at 25 °C and 75% relative humidity using a W3/031 water vapor permeability tester (Labthink Instrument, China). Weight changes were recorded every 15 min for 4 h.

#### 2.3.5. Wetting

The wettability of the coating films was assessed using a contact angle meter (OCA15EC, Data Physics Instruments, Filderstadt, Germany) through static contact angle measurements by dropping 2.0 μL of deionized water onto a slide and recording the measurements at three distinct positions.

#### 2.3.6. Release of OG in the Coating

Following the described method [20], the release property of OG from the K-P-OG coating was evaluated during storage. The absorbance of each sample was measured in triplicate at 296 nm. These measurements were recorded every three days for 30 days.

### 2.4. Antioxidant Activity, In Vitro and In Vivo Antifungal Activity Assay

The DPPH radical-scavenging activity of the coating solution was quantified using a previously described method [21]. The diameter of the inhibition zone (DIZ) was determined using an experimental method [22]. The DIZ was measured using a digital caliper. Each experiment was conducted in triplicate.

Sterilized *Mandarin oranges* were wounded at a depth of 5 mm on the equatorial side using a sterile puncher. Each wound was injected with 10 µL of a *Penicillium italicum* spore suspension (1 × 10^5^ CFU/mL). Following complete absorption of the spore suspension, the treated samples were immersed in sterile water, KGM (1 wt%), KP (1 wt%, KGM: *high-methoxy pectin* = 6:4), and K-P-OG 1.5% film-forming solution for 2 min before removal. After air-drying at ambient temperature, all samples were stored in a plastic fresh-keeping box at 25 ± 1 °C and 90 ± 1% relative humidity (RH). The lesion diameter and disease severity of both the treated and control fruit wounds were observed [23]. Each experiment was conducted in triplicate.

### 2.5. Preservation Effect on Mandarin orange

#### 2.5.1. Coating Treatment of *Mandarin oranges*

Fresh-picked *Mandarin oranges* were selected and subsequently disinfected using sodium hypochlorite (0.02%). The samples were then randomly allocated into four groups: a control group (CN, no coating treatment) and experimental groups (KGM, K-P-OG 0%, and K-P-OG 1.5% coating treatment). Each sample was immersed in the coating solution for 60 s. After natural drying, the samples were stored in a plastic fresh-keeping box at 25 ± 1 °C and a relative humidity of 90 ± 1%. The efficacy of the coating in preserving the samples was evaluated during the storage period of the study.

#### 2.5.2. Weight Loss and Firmness

*Mandarin oranges* were weighed at two-day intervals. Weight loss was assessed. The firmness (g) of the *oranges* was assessed using a texture analyzer (TA). XT plus, Stable Micro System Co., Ltd., Surrey, UK). A p/2 probe with a diameter of 2 mm was used to penetrate the *oranges* to a depth of 10 mm at a rate of 1.00 mm/s. Eight penetration sites were randomly selected along the equatorial region of each *orange*.

#### 2.5.3. Total Solid Content and Total Acid

The total solid content and total acidity were quantified using a sugar acid analyzer (ATAGO Ltd., Niigata, Japan). To test the total acid, the pristine juice was diluted 50 times. To assess the total acidity, the original juice was diluted by a factor of 50. Prior to measurement, the instrument was calibrated with water, and both indicators were expressed as percentage values.

#### 2.5.4. Vitamin C Content

The vitamin C (Vc) content in *Mandarin oranges* (mg/100 g) was quantified using a UV-Vis spectrophotometer (TU-1900, Beijing, China) following a previously described method with slight modifications [24].

#### 2.5.5. Peroxidase

The sample (5.0 g of *mandarin orange* tissue) was homogenized in 5 mL extraction buffer and centrifuged at 10,000 rpm for 30 min at 4 °C. Subsequently; the supernatant was collected and combined with 3 mL of guaiacol solution and 200 µL of H_2_O_2_ solution. The POD activity in the pericarp was determined and calculated following a previously described method with slight modifications [25], and the results were described as ∆OD_470_ min^−1^·g^−1^.

#### 2.5.6. Decay Rate

Every two days, a random selection of 20 *oranges* was made from each group for testing, and the physiological indices of the samples were assessed visually. Each measurement was conducted in triplicate, and the mean values were calculated. The decay rate was evaluated as follows:

(1) $$Decay\;rate=\frac{Number\;of\;rotted\;oranges}{Total\;number\;of\;oranges}\times 100\%$$

## 3. Results

### 3.1. Stability of the Coating Solutions

**Figure 1:**
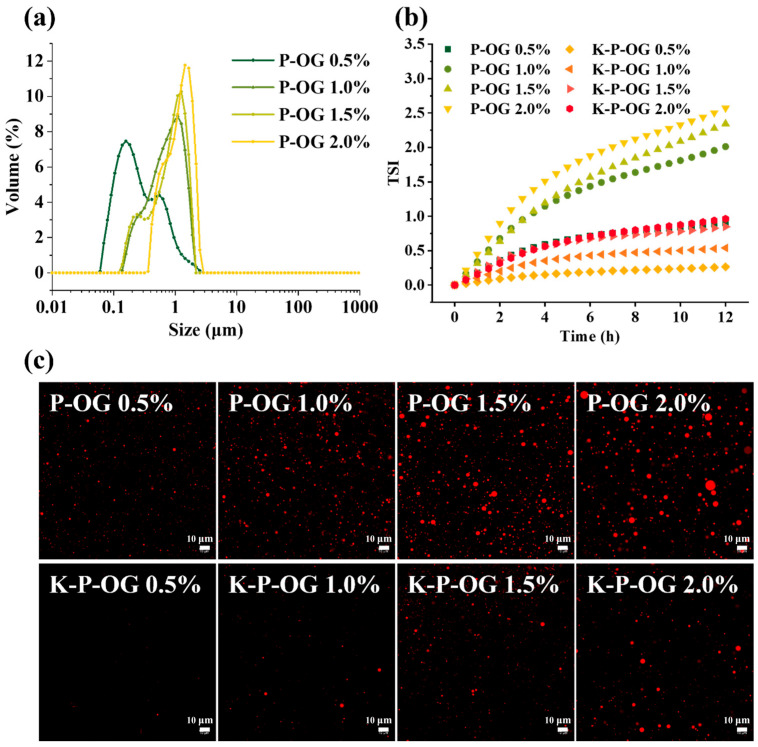
Droplet size distribution (**a**) and microstructure (**c**) of the emulsion and film-forming emulsions loaded with varying concentrations of OG at 25 °C. TSI values (**b**) of emulsions and film-forming emulsions within 12 h at 25 °C.

The distribution of droplet sizes in the coating solutions was assessed using a laser particle sizer, as illustrated in Figure 1a. The average D_[2,3]_ droplet size of the P-OG emulsion coating solutions was 0.35–1.08 µm with a narrow peak distribution, indicating that high-methoxyl pectin could effectively incorporate OG. The hydrophobic phenolic fraction of high-methoxyl pectin facilitated adsorption onto the OG droplet, whereas the hydrophilic fraction served as an effective steric stabilizer [26,27]. Additionally, the droplet size distribution of the OG emulsions loaded with 0.5% OG exhibited a bimodal peak due to unabsorbed pectin. As the concentration of OG increased, the droplet size of the emulsions (Table 1) gradually increased, indicating that flocculation or aggregation occurred due to the weakening of the steric hindrance effect with a thinner interfacial layer [28]. CLSM’s optical micrograph of CLSM (Figure 1c) confirmed that the emulsion droplets were spherical and uniformly distributed.

TSI is a key parameter used to quantify emulsion stability. Unstable emulsions can result in an uneven distribution of ingredients, causing a reduction in the barrier properties of the coatings. As the OG concentration increased, the TSI values of the P-OG and K-P-OG emulsions gradually increased (Figure 1b). It was found that the K-P-OG coating solution had high stability, with a TSI value between 0.2~0.5, which was obviously lower than that of the P-OG emulsions (1.8~2.5). The high viscosity of KGM prevented emulsion flocculation and coalescence, significantly improving emulsion stability. In conclusion, the K-P-OG coating exhibited high uniformity and stability, which facilitated a homogeneous microstructure within the coating, thereby significantly enhancing its gas barrier properties and protective performance.

### 3.2. Apparent Viscosity Analysis

**Figure 2:**
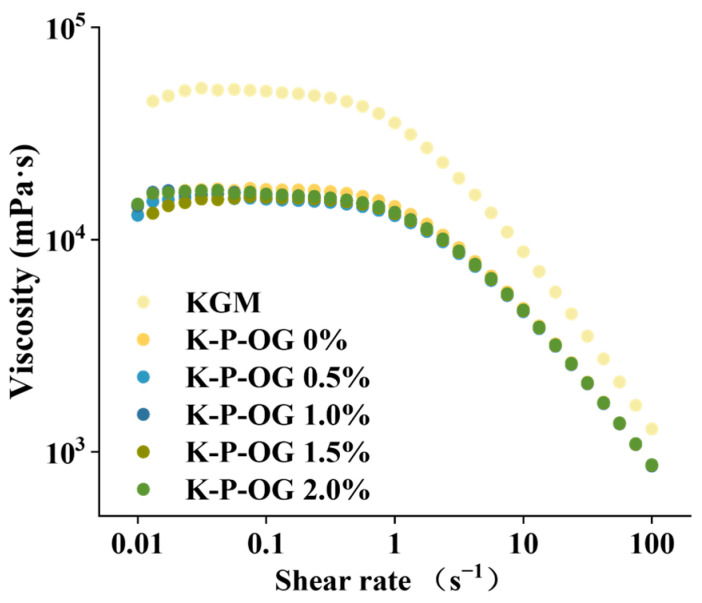
Viscosity of emulsions and film-forming emulsions at 25 °C.

The apparent viscosity is essential for improving the adhesion of fruit peel coatings [29]. The apparent viscosity curve of the K-P-OG coating solution is shown in Figure 2. When external forces were applied, all the coating solutions displayed shear-thinning behavior, which was attributable to the rapid destabilization of the polymer network [30]. The zero-shear viscosity of the KGM dispersion was 50 Pa·s. However, the addition of the P-OG emulsion decreased the zero-shear viscosity (16 Pa·s). This could be attributed to the P-OG emulsions diluting the KGM dispersion by disrupting the structure of the KGM network, thus reducing KGM macromolecular entanglement. Similar results have been reported for KGM-based film-forming liquids loaded with oregano essential oil stabilized by zein-pectin nanoparticles [31]. In the K-P-OG coating solution, the high viscosity of KGM significantly contributed to the pseudoplastic behavior of the coating solutions, whereas the inclusion of P-OG emulsions enhanced the flowability of the coating, thus providing good adhesion to fruit peel for edible coating preservation applications [12]. Furthermore, it has been reported that incorporating high-methoxyl pectin as an emulsifier in edible coatings enhances the adhesion of the coating to the fruit surface. This improvement is attributed to pectin’s ability to provide wettability by preventing lipid migration and reducing the surface tension at the water-lipid interface [32]. Consequently, the K-P-OG coating achieved an optimal viscosity, which facilitated its adhesion to the fruit surfaces.

### 3.3. SEM

**Figure 3:**
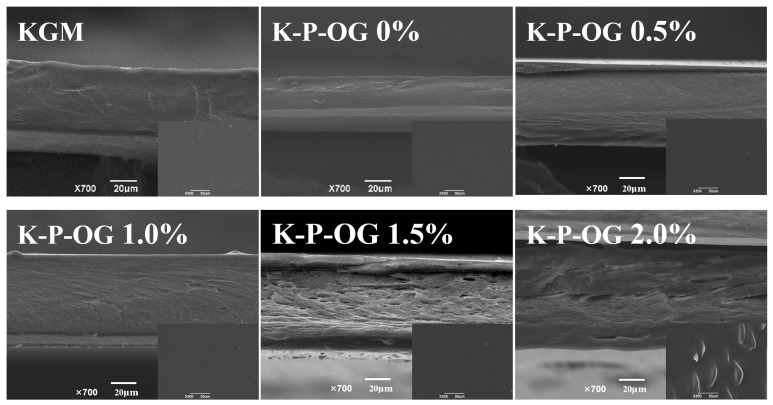
The scanning electron microscopy (SEM) micrographs of the surface and cross-section of the films.

As shown in Figure 3, the surfaces of the KGM films were smooth, dense, and homogeneous, consistent with the results of a previous study [12]. In comparison to the KGM film, the cross-section of the KP film exhibited a denser and smoother network structure. This observation confirms the strong compatibility between KGM and pectin, which is attributed to the hydrogen bonding between them [19]. Furthermore, OG was distributed in the network of the polysaccharide matrix, suggesting that OG was successfully emulsified and was very stable during the dry process. The dense and compact structure with a homogeneous distribution of OG droplets provides good mechanical and air barrier properties. However, the surface of the K-P-OG 2.0% group appeared as a rough film surface with a discontinuous structure and tiny pores. This phenomenon was associated with reduced stability due to excess OG, which was consistent with the TSI and CLSM results. This phenomenon was associated with reduced stability due to excess OG, aligning with the findings from CLSM and TSI value analyses. During evaporation, the droplets tended to aggregate, which subsequently weakened the polysaccharide interactions within the film network and disrupted the continuity of the KGM-based coating film structure [33]. The addition of the P-OG emulsion to the KGM matrix produced a more complex network structure, which significantly altered the physicochemical properties.

### 3.4. Air Barrier Properties

**Figure 4:**
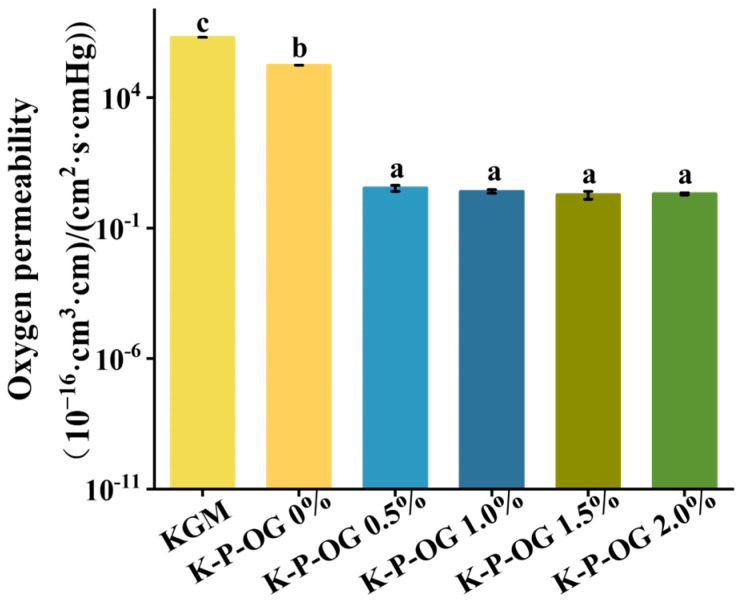
Oxygen permeability (OP) of the films at 25 °C. Different superscript letters within the same column indicate significant differences among the formulations (*p* < 0.05).

Oxygen permeability (OP) is a critical factor in edible membranes, as it influences food oxidation and degradation of food quality [34]. The OP of KGM, KP, and K-P- is shown in Figure 4. Compared to the KGM film (1.99 × 10^−10^ cm^3^·cm/cm^2^·s·cmHg), the KP films exhibited lower OP (1.73 × 10^−11^ cm^3^·cm/cm^2^·s·cmHg) due to the compacted structure of the KP film, which blocked or lengthened the oxygen entry pathways [35]. The incorporation of OG significantly enhanced the oxygen barrier properties of the KP films by six orders of magnitude. This improvement may be attributed to several factors, including the following. Notably, the OG droplets were uniformly distributed within the film matrix, maintaining the integrity of the biopolymer network structure. This provided an extremely tortuous pathway for oxygen to penetrate the film, which significantly reduced the OP [36]. In contrast, pectin effectively encapsulated OG and KGM with high viscosity, further stabilizing the coating solution. The stable system avoided the volatilization of OG, which would provide oxygen permeation channels [37]. In addition, the low oxygen solubility and antioxidant compounds of OG also contributed to enhancing the oxygen barrier properties of the coating film [38].

**Figure 5:**
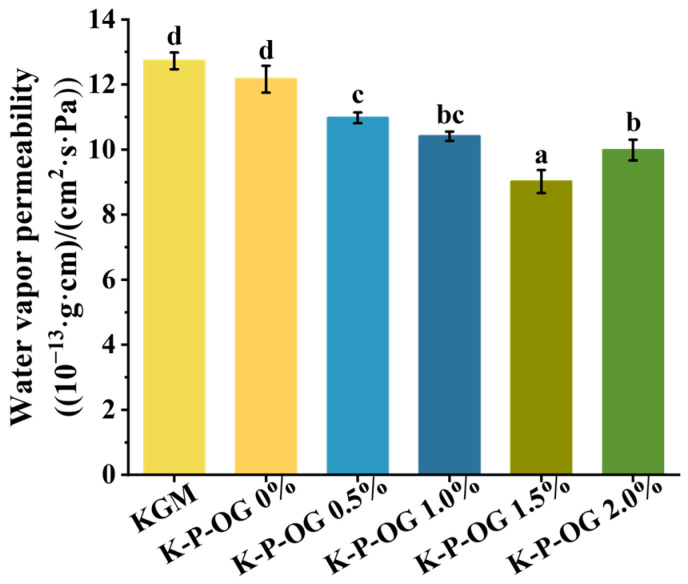
Water Vapor Permeability (WVP) of the films at 25 °C. Different superscript letters within the same column indicate significant differences among formulations (*p* < 0.05).

The permeation mechanism of biopolymers is based on their adsorption, diffusion, and desorption. With regards to Water Vapor Permeability (WVP), it mainly depends on the hydrophilicity of the material and the network structure within the biopolymer matrix [39]. Food packaging that exhibits superior water vapor barrier properties can effectively inhibit the transfer of moisture between food products and their surrounding environment [12]. As shown in Figure 5, the WVP of the KGM film was 12.73 × (10^−13^ g·cm)/(cm^2^·s·Pa). The addition of P-OG emulsions (0.5–1.5%) decreased the WVP of the KGM-based films. The 1.5% OG-loaded film had the maximum water vapor barrier (9.02 × (10^−13^ g·cm)/(cm^2^·s·Pa)), indicating its highest water barrier properties. The superior performance was attributed to the uniform distribution of the OG emulsion, which created a dense tortuous path for moisture diffusion and improved the hydrophobicity of the coating film. Moreover, the compact structure of the coating films, formed by the hydrogen-bonded network between the hydroxyl group of KGM and the carboxyl group of pectin, improved the water vapor barrier performance [40]. When the OG concentration was up to 2.0%, the WVP increased to 9.98 × (10^−13^ g·cm)/(cm^2^·s·Pa). The disruption of the dense structure of the film was attributed to the presence of larger droplets of P-OG, which allowed the penetration of water vapor through the film [41]. These findings indicate that K-P-OG may serve as an effective material for moisture-resistant food packaging.

### 3.5. Wetting Property

**Figure 6:**
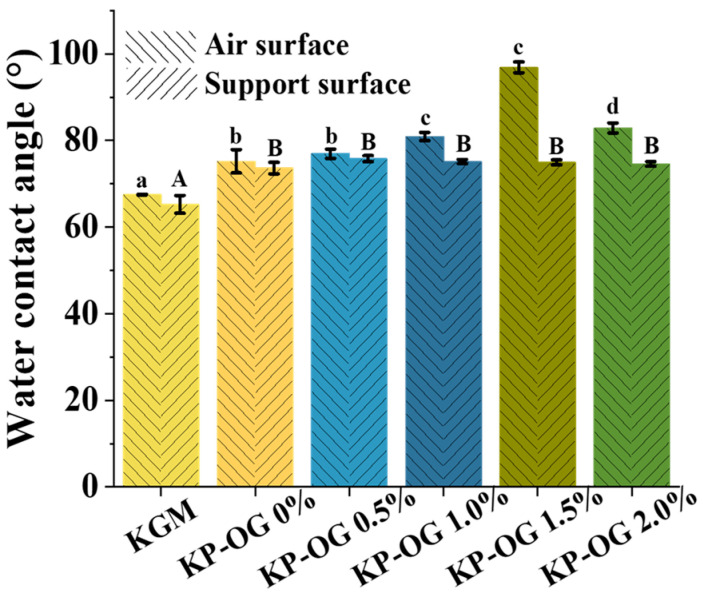
Water contact angle (WCA) of the films at 25 °C. Different superscript letters within the same column indicate significant differences among formulations (*p* < 0.05).

The water contact angle (WCA) is widely used to evaluate the wetting behavior of a liquid on a solid surface [42]. The WCA of the sample fruits is shown in Figure 6. The KGM film exhibited the lowest initial water contact angle values, measuring 67.5° on the air surface and 65.3° on the support surface, thereby demonstrating the hydrophilic nature of KGM. In contrast, the KP films displayed higher water contact angle values due to their more compact structures [43]. The water contact angle (WCA) values at the air interface of the film increased with the concentration of OG. This phenomenon is attributed to OG’s ability to reduce the exposure of hydrophilic groups on the surface of the coating film. However, the WCA value on the air surface of the films decreased from 96.8° (K-P-OG 1.5%) to 82.9° (K-P-OG 2.0%), which was due to the destabilization of emulsion droplets during the coating drying process [44]. This was consistent with the SEM observations. The WCA value of the air surface was higher than 90°, providing a water barrier for the coating, while that of the supporting surface was approximately 75°, facilitating the wetting of the coating on the fruit surface. Consequently, the incorporation of the OG emulsion effectively increased the WCA, thereby enhancing the wettability of the K-P-OG coating on the fruit.

### 3.6. The Release Performance of OG from the Coating

**Figure 7:**
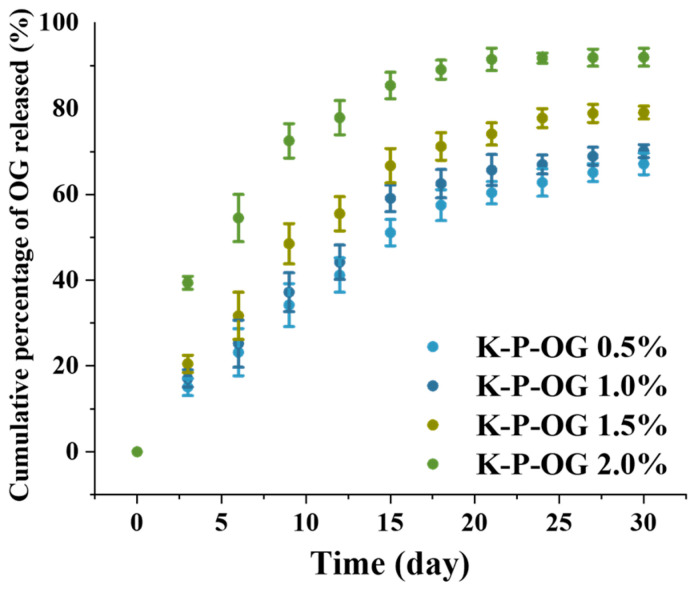
Cumulative percentage of OG released over time at 25 ± 1 °C and 55 ± 5% RH.

The release performance of the OG from the coating during storage at room temperature is shown in Figure 7. Initially, OG was released at a relatively rapid rate, followed by a slower and more sustained release. As the storage duration increased, OG gradually diffused into the interior of the coating due to the progressive weakening of the interaction between pectin and OG. Consequently, as the concentration gradient between the OG in the coating and the external atmosphere diminishes over time, the release rate of the OG decreases [20].

At day 29, the cumulative release of K-P-OG 0.5% was 60%, which was much lower than that of the K-P-OG 2% coating due to its varied stability, as indicated by the TSI results. The release retention of the K-P-OG 1.5% coating solution was 52% on day 12. This finding indicates that the system may significantly contribute to the sustained release of OG from the dense interfacial layer of high-methoxyl pectin, as well as from the compact structure of the K-P-OG coating solution. By adding P-OG emulsion, OG was slowly released from the KGM matrix, thereby enhancing its antifungal, antioxidant, and food preservation properties.

### 3.7. Antioxidant Activity

**Figure 8:**
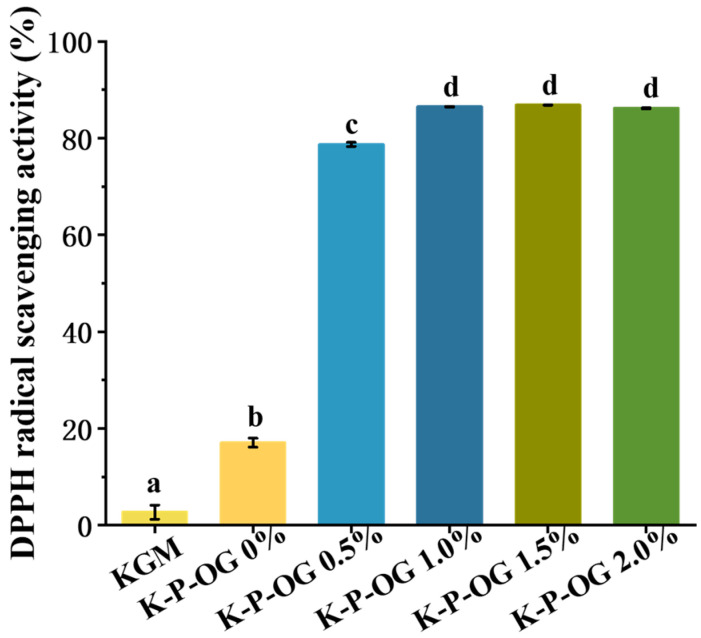
DPPH radical-scavenging activity of the films. Different superscript letters within the same column indicate significant differences among formulations (*p* < 0.05).

The greater the hydrogen supply capacity of an antioxidant, the more effective it is at scavenging DPPH radicals [45,46]. As shown in Figure 8, the KGM coating had the lowest antioxidant activity (2.68%) because KGM has a limited ability to provide hydrogen for scavenging free radicals [45]. Owing to the antioxidant properties of pectin, the KP film exhibited enhanced antioxidant activity (17%) [47]. The incorporation of OG into the coating substantially enhanced its antioxidant activity, ranging from 78.7% to 86.8%. This improvement is attributed to eugenol, the predominant constituent of OG, which functions as a primary antioxidant by scavenging and inactivating free radicals produced during oxidation. Furthermore, the presence of other components, such as limonene and linalool, enhances the antioxidant activity of the coatings [48,49]. These findings confirmed that OG enhanced the antioxidant activity of the coating and inhibitedfruit oxidation.

### 3.8. In Vitro and In Vivo Antifungal Test

**Figure 9:**
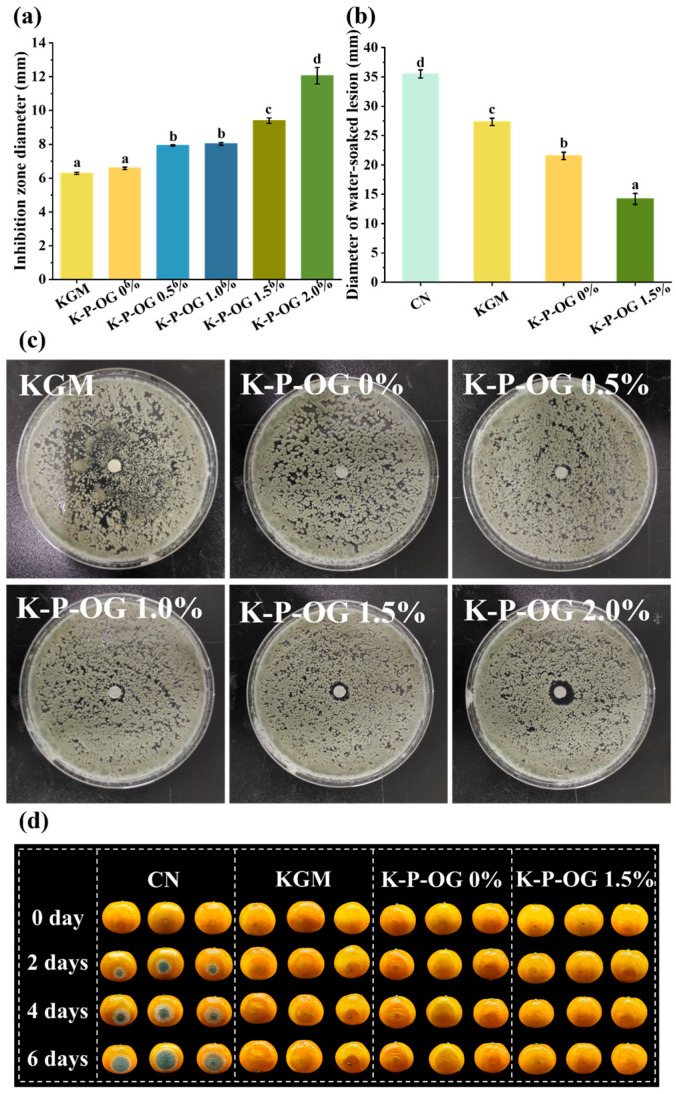
*P. italicum* inhibition zone diameters (**a**) and inoculation photos (**c**) of the film. Inhibition effect (**b**) and diameter (**d**) of the films against *P. italicum* in vivo. Different superscript letters within the same column indicate significant differences among formulations (*p* < 0.05).

*Oranges* are highly susceptible to *Penicillium* during storage and preservation [50]. The antifungal activity of the coatings was assayed against *Penicillium* by evaluating the inhibition zone diameter (Figure 9a). The KGM and KP coatings had almost no inhibitory effect on *Penicillium italicum* (Figure 9c). With the addition of OG, the diameter of the inhibition zone gradually increased from 6.28 ± 0.06 mm to 12.06 ± 0.50 mm. This observation confirmed that OG, serving as an antibacterial agent, enhanced the antimicrobial efficacy of the coatings. This is because eugenol (4-allyl-2-methoxyphenol), the main active compound (82.4%) in OG, impaired fungal membrane integrity and morphology due to its lipophilic character, which caused the overflow of intracellular ions and biomolecules, as well as morphological degeneration of *Penicillium italicum* [49,51,52,53]. Furthermore, eugenol may cause structural damage to the mitochondrial membrane of fungal spores, disturb mitochondrial function, and affect metabolic dysfunction [54]. In addition, it can inhibit the biosynthesis of fungal ergosterol, thereby compromising the structural integrity and function of the cell membrane. Eugenol has been reported to reduce the utilization of carbon sources and glucose uptake by fungi [55]. In vitro microbiological assays on *Penicillium italicum* demonstrated that the K-P-OG coating effectively inhibited growth in a concentration-dependent manner, rendering it suitable for *orange* preservation.

To evaluate the impact of the treatment on inhibiting spore germination and proliferation on the peel surface, *Mandarin oranges* were inoculated with *Penicillium italicum* and treated with KGM, KP, and K-P-OG, and the symptoms of fruit decay were recorded. As shown in Figure 9b, the treatment suppressed the growth of *Penicillium italicum* and inhibited lesion diameter. All fruits in the control group exhibited disease symptoms. On day 0, a small amount of white mycelium was observed around the wounds (Figure 9d), which penetrated deeply into the infected tissues and developed into large blue conidia (35 mm). In the KGM coating group, white mycelium was observed in only one of the samples from day 2, but it had not formed citrus-green mold. In addition, water-soaked lesions appeared on the surface, and the edge of the wound was in a state of soft rot, with a diameter of 27 mm on day 6. This was attributed to the destruction of the peel tissue and dysfunction of the cells in the epidermal layer of citrus by *Penicillium italicum* [56]. The KP coating demonstrated a superior inhibitory effect on *Penicillium italicum* compared to KGM, as it only had watery lesions around the wounds without the development of white mycelium or blue conidia. With the addition of OG, the fruit samples exhibited the lowest infection rate by the sixth day, and the extent of soft rot in proximity to the infection site was significantly reduced (13 mm). The results indicated that the K-P-OG coating had an obvious inhibitory effect on *Penicillium italicum*. In vivo experiments further substantiated that the K-P-OG coating played an important role in the virulence of *Penicillium italicum*, thereby enhancing the fruit’s disease resistance and reducing the incidence of postharvest fungal infections. The application of the K-P-OG coating effectively extended its antibacterial efficacy and improved its overall performance.

### 3.9. Preservation Effect

**Figure 10:**
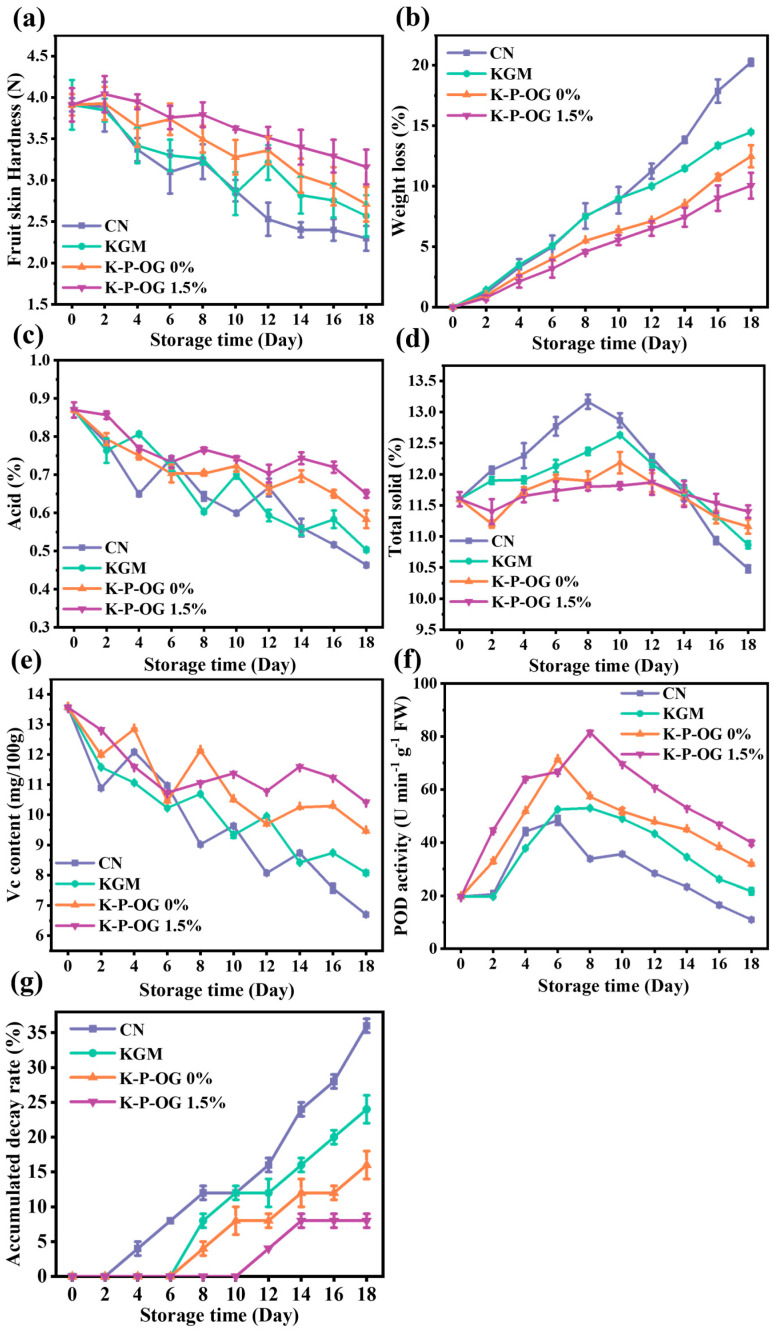
The weight loss (**a**), firmness (**b**), total acid (**c**), total solid (**d**), Vc content (**e**), POD activity (**f**), and accumulated decay rate (**g**) of citrous fruits during storage.

To verify the feasibility of the K-P-OG coating effect, firmness, weight total acids (TA), total solid content (TSS), peroxidase (POD), and decay rate were measured during storage and are presented in Figure 10. After harvest, fruits continue to undergo respiration, a metabolic process in which they absorb oxygen and subsequently release carbon dioxide and water as the end products. During ripening, the respiration rate increases continuously until it reaches the climacteric peak [57]. Fruit firmness reflects freshness and ripeness. As fruit ripens, the cell wall is degraded by enzymes, leading to the loss of firmness [58]. Figure 10a shows the changes in the firmness of the coated *Mandarin oranges* during storage. The control, KGM, KP, and K-P-OG 1.5% coating groups all exhibited a decreasing trend, with a reduction in firmness of 41%, 36%, 30%, and 18%, respectively. The gas barrier properties of the edible coating suppressed respiration by restricting the amount of oxygen absorbed by the fruit, resulting in a varied respiration rate. The K-P-OG 1.5% group showed the slowest decline in firmness, with firmness of 3.16 N on day 18, which was nearly half of that in the control group. This was attributed to the fact that both the gas barrier and antioxidant activity of the K-P-OG coating contributed to maintaining the firmness of the *Mandarin oranges* by limiting oxygen permeation and preventing water loss from the epidermal cells of the fruits [59].

As shown in Figure 10b, the weight loss of all samples increased with storage duration. As soon as the fruit is harvested, the fruits stop obtaining water and begin to lose moisture through transpiration. Approximately 97% of the fruit weight is lost due to moisture loss. On day 18, the control group exhibited the most significant weight loss (21%) due to the direct exposure of uncoated *oranges* to environmental conditions. In contrast, the coatings served as an effective barrier on the fruit epicarp, sealing cracks and pores and covering stomata and lenticels, thereby impeding the respiration process and metabolic reactions. The incorporation of lipids into the coating enhances its hydrophobicity, which has been reported to improve its effectiveness. The K-P-OG coating had the lowest weight loss (7.5%) because OG, as a hydrophobicity-imparting substance, enhanced the effect of reducing moisture. The K-P-OG coating reduced the loss of nutrients by 13.5%, inhibiting microbial growth. The results indicated that a higher degree of weight loss corresponded to a reduction in firmness, which is consistent with the results of the in vivo antifungal assessment. The smallest weight loss was observed in the K-P-OG 1.5% group, which was the most suitable for *orange* preservation.

Total acids (TA) in fruits are generally transformed into sugars or serve as substrates for respiratory processes [60]. The acidity of all coating groups gradually decreased during storage (Figure 10c). As an end product of carbohydrate metabolism, organic acids are consumed by respiration. On day 18, the TA of the coating group, especially the K-P-OG group (0.78), was significantly higher than that of the control group (0.45). The findings confirmed that the coating as an effective barrier slowed the respiration rate and transpiration of fruits, thereby decelerating the conversion of organic acids to sugars [25]. In addition, OG, as an antimicrobial agent also contributed to inhibiting the respiration of *oranges* [56]. As shown in Figure 10d, the TSS values of the samples first increased and then decreased. At the initial stage, carbohydrates were converted into sugars and other soluble compounds by catabolic processes, which resulted in an increase in the TTS value. The application of coating groups significantly mitigated the increase in total soluble solids (TSS) content during storage, primarily due to the suppression of respiration. The later decrease in TSS value was due to the fact that soluble sugars were consumed by respiration during the metabolism process [56,61]. At the end of the storage period, the TSS content in the control group was reduced to 10.2%, while the TSS content of fruit coated with K-P-OG remained constant, as the coating inhibited the respiration and metabolism of the fruit and slowed down the aging process [62].

As an antioxidant, vitamin C (Vc) in *oranges* plays a crucial role in neutralizing free radicals, including hydroxyl radicals, superoxide anions, and hydrogen peroxide, through the vitamin C peroxidase reaction. This mechanism is essential for preventing the oxidative degradation of fresh fruits during ripening [63]. As shown in Figure 10e, the Vc content in *oranges* decreased during storage. This is related to the physiological effects of fruits and the influence of microorganisms. Notably, the control group demonstrated the most rapid decline, whereas the K-P-OG 1.5%-treated group showed the slowest decrease. In general, a higher vitamin C retention corresponds to a lower OP. The KGM group coatings blocked oxygen to a certain extent, reduced the respiration rate, inhibited microbial growth, and thus reduced the loss of Vc content. As a natural hydrophobic antioxidant, it could further reduce gas exchange with the external environment and inhibit physiological reactions, thus preventing VC oxidation. Consequently, the K-P-OG 1.5% group exhibited the highest Vc content, thereby effectively preserving the nutritional quality of the *oranges*.

Peroxidase (POD) is the principal antioxidant enzyme that modulates the levels of reactive oxygen species in plants [25]. As shown in Figure 10f, the peroxidase (POD) activity in *Mandarin oranges* increased during the initial phase of storage, followed by a rapid decline in the later stages. Similar findings have been reported previously [64]. On day 18, the POD activities of KGM, KP, and K-P-OG treatments increased by 1.3, 1.7, and 2.1 times, respectively. The results revealed that the application of the K-P-OG coating substantially enhanced POD activity in the fruit during storage.

**Figure 11:**
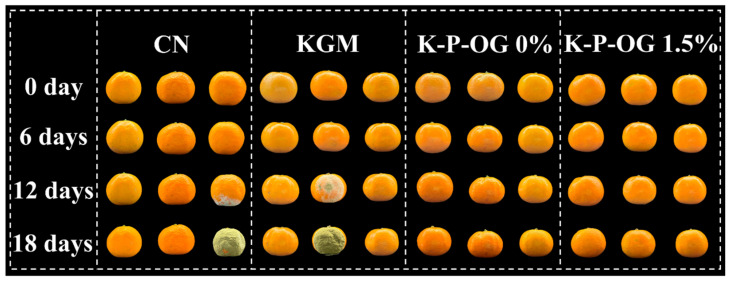
Exterior photos of citrous fruits during storage at 25 °C.

The microbial degradation of *Mandarin oranges* is mainly attributed to *Penicillium italicum*, *Penicillium digitatum*, and Rhizopus stolonifer, which are responsible for the development of green/blue mold and soft rot, respectively [65]. Delaying fruit decay is the main purpose of preservation (Figure 11). As displayed in Figure 10g, the uncoated samples started to decompose on day 2, with the rate of decay reaching 38% by day 18. In contrast, the sample treated with KGM and KP was infected on day 6, suggesting that the coating effectively reduced fruit exposure to the external environment and delayed decay time [66]. The sample covered with K-P-OG 1.5% remained in good condition with no fungal infection within the first 10 days, as OG further enhanced the barrier property and antifungal and antioxidant capacity of the coating. The decay rate of the K-P-OG 1.5% group was 7.1% on day 18, which was 30% lower than that of the uncoated group. The results demonstrated that the K-P-OG 1.5% coating effectively inhibited cell growth and prevented the contamination of external fungi, thus extending the shelf life for at least 8 days. Therefore, the K-P-OG edible coating application is promising for prolonging the shelf life of *Mandarin oranges* by delaying the ripening process and reducing quality losses.

## 4. Conclusions

This study presents the development of a simple yet effective emulsion coating specifically designed for the postharvest treatment of *Mandarin oranges*. As an alternative natural preservation method, a KGM-based coating incorporated with OG was prepared and assessed for its antifungal efficacy. The K-P-OG coating exhibited high uniformity and stability, as well as optimal viscosity, demonstrating superior performance in terms of flexibility, stability, and adhesion. Consequently, the incorporation of OG emulsion effectively increased the WCA value, thereby enhancing the wettability of K-P-OG coating on the fruits. The incorporation of the OG emulsion effectively increased the WCA. The WCA value of the air surface was higher than 90°, providing a water barrier for the coating, while that of the supporting surface was approximately 75°, facilitating the wetting of the coating on the fruit surface, thereby enhancing the wettability of the K-P-OG coating on the fruits. The compatibility between KGM, pectin, and OG contributed to a highly compact and homogeneous structure, which favored its air permeability (maximum water vapor barrier: 9.02 × (10^−13^ g·cm)/(cm^2^·s·Pa), oxygen permeability: 7.9 × (10^−16^ g·cm)/(cm^2^·s·Pa)), thereby regulating postharvest respiration and enhancing postharvest storability, while simultaneously serving as a protective barrier against external microorganisms. Compared with the control group, the weight loss rate during storage of K-P-OG 1.5% was reduced by 13%, and firmness and POD were increased by 24.14% and 100%, respectively. This work presents a green and promising strategy using OG to protect *Mandarin oranges* against postharvest fungal decay and prolong their shelf life, aiming to reduce plastic pollution and food spoilage.

## Figures and Tables

**Table 1:** The average droplet size of emulsions with different OG concentrations.

Sample	D_[3,4]_ (μm)	D_[2,3]_ (μm)	Span
P-OG 0.5%	0.48 ± 0.22 ^a^	0.35 ± 0.29 ^a^	3.11 ± 0.19 ^c^
P-OG 1.0%	0.75 ± 0.03 ^b^	0.51 ± 0.04 ^b^	1.61 ± 0.10 ^b^
P-OG 1.5%	0.86 ± 0.04 ^b^	0.55 ± 0.02 ^b^	1.49 ± 0.02 ^b^
P-OG 2.0%	1.21 ± 0.07 ^c^	1.08 ± 0.15 ^c^	0.83 ± 0.31 ^a^

^a–c^ Different superscript letters within the same column indicate significant differences among the formulations (*p* < 0.05).

## Data Availability

Data are contained within the article.
